# LncRNA SNHG5 promotes the proliferation and cancer stem cell-like properties of HCC by regulating UPF1 and Wnt-signaling pathway

**DOI:** 10.1038/s41417-022-00456-3

**Published:** 2022-03-25

**Authors:** Yarui Li, Junbi Hu, Dan Guo, Wenhui Ma, Xu Zhang, Zhiyong Zhang, Guifang Lu, Shuixiang He

**Affiliations:** Department of Gastroenterology, the First Affiliated Hospital of Xi’an Jiaotong University, Xi’an, Shaanxi 710061 P.R. China

**Keywords:** Cell biology, Cancer stem cells, RNAi, Cancer genetics

## Abstract

The role of long noncoding RNA (lncRNAs) had been demonstrated in different types of cancer, including hepatocellular carcinoma. This study was intended to investigate the role of lncRNA small nucleolar RNA host gene 5 (SNHG5) in HCC proliferation and the liver CSC-like properties. Through functional experiments, we determined that knockdown of SNHG5 repressed HCC cell proliferation and CSC-like properties, while over-expression of SNHG5 promoted cell growth. At the same time, CSC markers (CD44, CD133, and ALDH1) and related transcription factors (OCT4, SOX2, and NANOG) were downregulated when SNHG5 was knocked down. Mechanically, RNA immunoprecipitation (RIP) and RNA pulldown assay showed that SNHG5 regulated the proliferation and CSC-like properties of HCC by binding UPF1. Further investigations showed that expression of critical components of Wnt/β-catenin pathway (β-catenin, TCF4, c-myc, cyclinD1, and c-Jun) were upregulated with depletion of UPF1 in liver CSCs, which were downregulated with depletion of SNHG5. After use of the inhibitor of Wnt/β-catenin pathway, the formation of liver CSCs sphere decreased. Taken together, SNHG5 plays a critical role to promote HCC cell proliferation and cancer stem cell-like properties via UPF1 and Wnt/β-catenin pathway.

## Introduction

Hepatocellular carcinoma (HCC) is the sixth common human malignancy in the world [1, 2]. Although the diagnosis and treatment techniques have improved significantly in recent years, the long-term survival rate among the HCC patients is still very low, this is mostly because of the fast metastatic property and higher rate of recurrence [3, 4]. Therefore, finding an alternate therapeutics and the identification of the underlying mechanism of progression is crucial in this situation.

Tumor cells characteristically present in a heterogeneous manner and thus influencing growth, metastasis, and recurrence. Heterogenicity might result from cells showing stem cell like character, otherwise known as cancer stem-cells (CSCs) [5]. CSCs have the abilities to self-renew, differentiate, and uncontrollable growth, that results in the formation of new growth in the local or a distant organ, which integrates with the non-CSCs [6–8], thus, contributing to the progression, metastasis, and recurrence of cancers. The existence of liver cancer stem cells (CSCs) is a known fact, and this group of cells has been characterized by several makers, such as CD133, CD13, CD90, and EpCAM [9–12].

Long noncoding RNA (lncRNA) length larger than 200nt does not possess the ability to encode proteins, thus remarked as “dark matter” in human diseases. LncRNAs have been demonstrated to regulate important biological functions, such as stem cell properties and tumor progression [13]. However, the functions and mechanisms of lncRNAs in CSCs are controversial. The small nucleolar RNA host gene 5 (SNHG5), one of the well-defined cytoplasmic lncRNAs, also known as U50HG, is 524 bp in length. Our previous research confirmed that SNHG5 was highly expressed in HCC tissues and was related to the prognosis of HCC patients, and further investigations showed that lncRNA SNHG5 plays a role in HCC metastasis [14]. Recently, many researches have revealed that lncRNAs have the ability to bind to DNA or RNA by a complementary sequence, which plays critical roles in mRNA splicing, RNA decay and translation, additionally, the post-translational modification of proteins can be modulated by lncRNAs [15, 16]. UPF1 is a key player in RNA-degradation pathways, and also essential for accomplishing DNA replication. Additionally, UPF1 interacts with many RNA substrates and promotes mRNA stability [17]. The important thing is that we find that UPF1 contains a potential binding site with SNHG5, which prompted our interest in investigating the biological roles and relationships of SNHG5 and UPF1 in HCC CSCs.

The present study aims to analyze the effect of SNHG5 on the proliferation and establish the cancer stem cell-like properties of HCC, explore the function of the SNHG5 in regulating the properties of HCC CSCs through UPF1 and Wnt-signaling pathway in vitro, and in promoting tumorigenesis in vivo.

## Materials and methods

### Cell lines and cell culture

The human HCC cell lines HepG2 and Huh7 were obtained from the Chinese Academy of Sciences Cell Bank (Shanghai, China). The cell lines have been tested and authenticated by short tandem repeat (STR).The HCC cells were cultured in the DMEM/High Glucose (Hyclone, USA) medium in a humidified incubator at 37 °C temperature and 5% CO_2_ concentration. About 10% FBS (fetal bovine serum, Gibco USA) and penicillin–streptomycin (100 U/mL and 100 μg/mL, respectively) were added in the DMEM/High Glucose medium prior to culture.

### Constructions of plasmid and cell transfection

SNHG5 and UPF1 overexpression plasmids, SNHG5-knockdown plasmids (SNHG5 shRNA with a corresponding negative control shRNA-NC), SNHG5-Mut/WT plasmids (pCMV-SNHG5- Mut vector containing mutations at the putative UPF1-binding site was generated by site-directed mutagenesis) were designed by Genechem (Shanghai, China). The GV248 vector was used, and the stable clones were selected by 5 μg/ml puromycin-containing medium. The puromycin-resistant cell clones were established after 4 weeks. Gene-expression level was evaluated by quantitative real-time PCR. The siRNA (small-interfering RNA) against UPF1 was designed by Genepharma (Shanghai, China). According to the manufacturer’s protocol, HCC cells were transfected with plasmid by using Lipofectamine 2000 (Invitrogen, Carlsbad, CA, USA).

### Cell-proliferation assays

#### MTT (3-[4, 5-dimethylthiazol-2-yl]-2,5-diphenyltetrazolium bromide) assay

MTT (0.5 mg/ml) was added into transfected cells and kept in the dark for 4 h. Then, the supernatant was removed, 150 μl of DMSO was added, and the optical density (OD) was measured at 490 nm.

#### EdU-incorporation assay

Transfected cells were seeded in to a 96-well plate (2 × 10^3^) embedded with complete growth medium. Then according to the manufacturer’s protocol, the process was carried out with EdU-detection kits (Keygen, Nanjing, China). The experiment was done in triplets. The cells were imaged with an inverted fluorescent microscope (Nikon Eclipse Ti-S) (20X).

#### Colony-formation assay

After routine incubation, transfected cells were trypsinized, centrifuged, counted, and replanted at a density of 500 cells/6 cm plate. After 12 days, the cell colonies (one colony containing at least 50 cells) were fixed with 37% methanol, stained with 0.1% crystal violet, and counted under a microscope.

### Sphere-formation assays

Ultra-low-attachment culture dishes (Corning, USA) were used to culture HepG2 and Huh7 cells with DMEM/F12 (Gibco, USA) added with 1% FBS, 20 ng/mL epithelial growth factor, and 20 ng/mL fibroblast growth factor for two weeks. The formation and the number of spheroids were detected by a stereomicroscope (Olympus, Japan).

### RNA isolation and quantitative real-time PCR

The total RNA was extracted from the cultured cells and the collected HCC tissues by using Trizol reagent (Invitrogen, Carlsbad, CA, USA) according to the manufacturer’s protocol. The Prime Script TM RT Master Mix Kit (Takara, Japan) and Mir-X miRNA qRT-PCR SYBR Kit (Takara, Japan) were used to obtain the cDNA. Quantitative real-time PCR (qRT-PCR) was performed with SYBR Premix Ex Taq™ II (Takara)on Thermal Cycler CFX6 System (Bio- Rad). β-actin as the endogenous control of qRT-PCR. The 2^−ΔΔCt^ method was used to calculate relative gene expression. Primer sequences for PCR were presented in Supplementary Table 1.

### Western blot analysis

The total protein from the cultured HCC cells and the tissue samples was isolated by RIPA (Beyotime, Haimen, China) supplemented with proteinase and phosphatase inhibitors. BCA detection kit (Keygen, Nanjing, China) was used for the quantification according to the manufacturer’s protocol. During electrophoresis, 5% gel for concentration and 10% for separation were used. The proteins were then transferred on a PVDF membrane (Merck Millipore) and were blocked by 5% nonfat milk for 1 h. Then, the PVDF membrane was incubated overnight at 4^0^C with the primary antibodies (Supplementary Table 2). On the next day the secondary antibody (Zhuangzhi Biology, China) was diluted in TBST in a 1:5000 ratio, and the membranes were re-incubated for 1 h. The protein bands were evaluated by ECL immunoblotting kit following the manufacturer’s protocol (Millipore, USA).

### Immunofluorescence (IF)

Cells were cultured on glass coverslips for 24 h, then fixed in 4% paraformaldehyde at room temperature for 15 min, and washed by PBS. The adherent cells were permeabilized using 0.5% Triton X-100, and blocked with 10% goat serum for 1 h. After that they were incubated with primary antibody at 4 °C overnight and secondary antibodies with an appropriate dilution. The cells were washed gently with PBS for 3 times, then the coverslips were stained with DAPI and imaged with an inverted fluorescent microscope (Nikon Eclipse Ti-S) (100X).

### Tumor formation in BALB/c nude mice

Four-week aged BALB/c nude mice were purchased from the Central Laboratory of Animal Science, Xi’an Jiaotong University, China. They were randomly divided into two groups, 5 mice in each group. The mice were kept under sterile specific pathogen-free (SPF) environment. A subcutaneous injection of 5 × 10^6^/200 μl HepG2 cells stably transfected with SNHG5–shRNA or NC-shRNA were given to each mice. The tumor formation was carefully observed every 4-day interval. Eight days following the injection, the palpable tumors were observed (blinded to the group allocation). Four weeks following the injection, the subcutaneous formatted tumor nodes were executed for further detection(cervical-dislocation method executed experimental animals). This study was done according to the Guide line for the “Care and Use of Laboratory Animals of the National Institutes of Health” and was approved by the Medical Ethics Committee of the Experimental Animal Center of Xi’an Jiaotong University.

### Subcellular fractionation

The cytoplasmic and nuclear fractions of HepG2 or Huh7 CSCs were isolated using the Nuclear/Cytosol Fractionation Kit (Cell Biolabs). The detailed steps of the experiment were executed according to the manufacturer’s protocol.Then, RIP–PCR was performed to detect the SNHG5 expression of cytoplasmic and nuclear.

### Immunoprecipitation (RIP)

Millipore EZ‐Magna RIP RNA Binding Protein Immunoprecipitation kit (Millipore) was applied to performed RIP assays according to the manufacturer’s protocol. Rabbit polyclonal IgG (Millipore) and antibodies to UPF1 (Abcam) were used in RIP assays. Then, RIP‐PCR was performed, and total RNA was used as input controls.

### RNA pull-down assay

The length of synthetic biotinylated SNHG5 was synthesized by Genechem (Shanghai, China). Biotin RNA Labeling Mix (Roche) was used to perform RNA pulldown experiment. First, biotinylated SNHG5 or SNHG5-Mut was incubated with cell-protein extractions (1 mg), which were then targeted with streptavidin beads (Invitrogen) and washed. The associated proteins were resolved by gel electrophoresis. Specific bands were excised and identified by western bolt.

### Statistical analysis

The cell- and molecular-biology experiments have been implemented three times. Data were expressed as mean ± SD. Statistical analysis was performed using SPSS 23.0 (IBM, SPSS, Chicago, IL, USA) and GraphPad Prism V7.0 and Student’s *t*-test and one-way ANOVA were done to analyze the results. A two-tailed *P* < 0.05 was considered as statistically significant and *P* < 0.01 was considered highly significant.

## Results

### The effect of SNHG5 on HCC cell proliferation in vitro

**Fig. 1:**
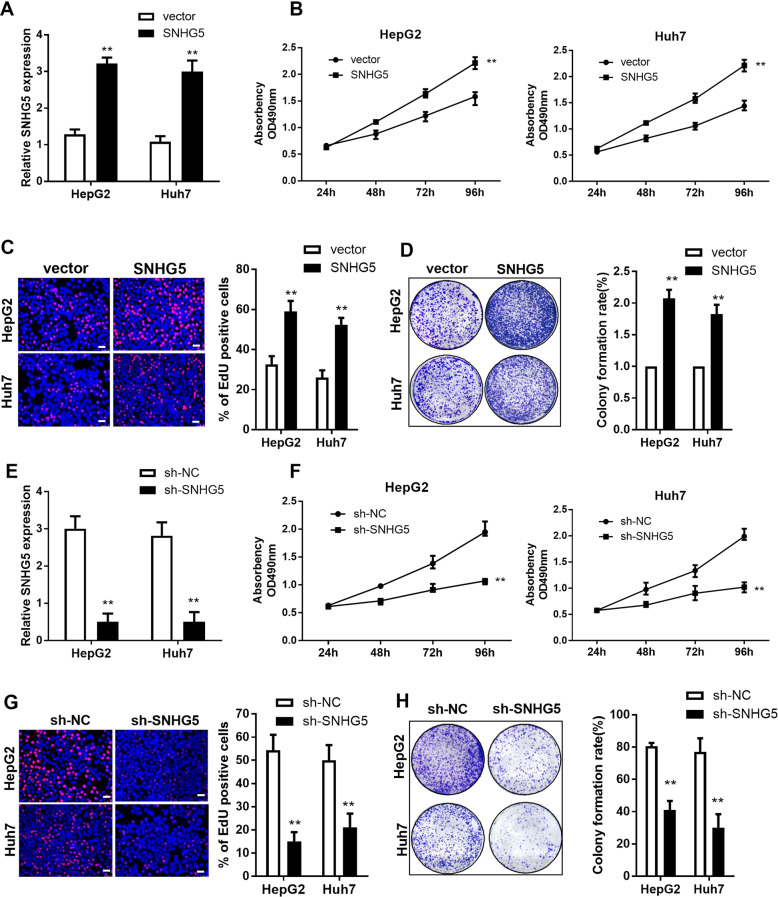
The effect of SNHG5 on HCC cell proliferation in vitro. **A** qRT-PCR analysis of SNHG5 expression after SNHG5 overexpression. MTT assays (**B**), EdU assay (**C**), and colony formation assay (**D**) showed that SNHG5 overexpression promotes HCC cells proliferation. (**E**) qRT-PCR analysis of SNHG5 expression after SNHG5 knockdown. MTT assays (**F**), EdU assay (**G**), and colony-formation assay (**H**) showed that downregulation of SNHG5 inhibits HCC cell proliferation. **compared with sh-NC or vector group *P* < 0.01. Scale bar: 20 μm.

The effect of SNHG5 on HCC cells in vitro was investigated by upregulation and knockdown of the expression of SNHG5. The results showed that the expression of SNHG5 dramatically increased after transfection with the pCMV–SNHG5 vector in HepG2 and Huh7 cells while compared with the empty vector (Fig. 1A). Cellular functional-validation experiments were performed in HepG2 and Huh7 cells, these included MTT-proliferation assay, colony-formation assay, and Edu assay. The MTT assay showed that overexpression of SNHG5 induced HepG2 and Huh7 cell proliferation in comparison with the control group (Fig. 1B). Upregulation of SNHG5 results in an elevated growth tendency of the HCC cells in the Edu and colony-formation assays (Fig. 1C, D). To further evaluate the role of SNHG5 on HCC cell proliferation, specific lentivirus-mediated short-hairpin RNA (shRNA) targeting SNHG5 were transfected into HepG2 and Huh7 cells, resulting in a significant decrease in SNHG5 expression (Fig. 1E). MTT, Edu assay and plate colony-formation assays showed that knockdown of SNHG5 inhibited the HepG2 and Huh7 proliferation compared with the control group (Fig. 1F–H). Moreover, we check for the recovery of the SNHG5 phenotype after shSNHG5 knockdown, the MTT assay showed that SNHG5 vector can restore the inhibition of sh-SNHG5 on cell proliferation (Fig. S1). All these results suggest that SNHG5 has a significant role in HCC cell proliferation.

### Knockdown of SNHG5 represses the liver CSC-like properties

**Fig. 2:**
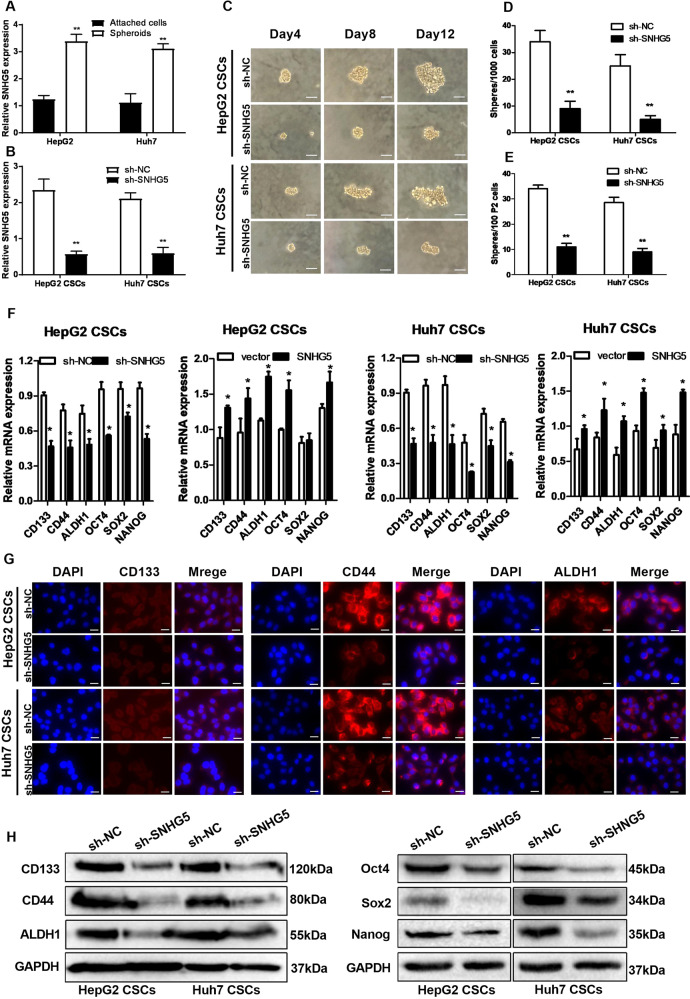
Knockdown of SNHG5 represses the liver CSC-like properties. **A** LncSNHG5 is upregulated in the spheroids compared with the attached cells derived from HepG2 and Huh7 cells. **B** qRT-PCR of SNHG5 expression in HepG2 and Huh7 CSCs by sh-NC and sh-SNHG5. **C** Bright-field microscopy images showed the typical morphological features of small aggregates and spheres after knockdown of SNHG5 in HCC CSCs separately on the 4th, 8th, or 12th day, white bar: 20 mm. **D**, **E** Quantification of the total number of primary spheres per 1000 single CSCs and secondary spheres (P2) per 100 single CSCs after SNHG5 knockdown. **F** qRT-PCR analysis of the expression of stem factors (SOX2, OCT4 and NANOG) and markers (CD133, CD44 and ALDH1) in SNHG5 Knockdown and overexpression of HepG2 and Huh7 CSCs. **G** Immunofluorescence (IF) analysis and (**H**) Western blotting analysis of the expression of stem factors and markers in sh-SNHG5 HepG2 and Huh7 CSCs compared with sh-NC cells. GAPDH as a loading control. *compared with sh-NC group <0.05, **P < 0.01. Scale bar: 50 μm.

Sphere formation is a selection method that enriches CSCs. To confirm the expression pattern of SNHG5 in liver CSCs, we enriched liver CSCs through inducing hepatoma spheroid formation and examined SNHG5 expression in the self-renewing spheroids and the attached cells. As shown in Fig. 2A, the SNHG5 expression was increased dramatically in liver CSCs. To analyze the role of SNHG5 in self-renewal characteristics of liver CSC, SNHG5 expression in HepG2 and Huh7 CSCs was reduced by SNHG5–shRNA (Fig. 2B). Then, the sphere-formation assays were performed to observe the number of primary spheres per 1000 single-liver CSCs and secondary spheres (P2) per 100 single-liver CSCs after SNHG5 knockdown. The adherent cells/spheroids were observed every 4-day interval. The result showed that the sphere-formation rate was dramatically impaired in SNHG5–shRNA cells compared with the sh-NC cells (Fig. 2C–E). We also examined the effects of SNHG5 on the expression of stem factors (Oct4, Sox2, and Nanog) and markers (CD133, CD44 and ALDH1) in two groups. The result of qRT-PCR showed that decreased SNHG5 expression inhibited the enrichment of these CSC markers in HepG2 and Huh7 CSCs, while overexpression of SNHG5 showed the opposite effect (Fig. 2F). IF (Fig. 2G) and Western blot analysis (Fig. 2H) reconfirmed that knockdown of SNHG5 inhibits the stemness of HCC CSCs. These results indicated the contribution of SNHG5 in liver CSC-like properties.

### SNHG5 regulates the expression of UPF1

**Fig. 3:**
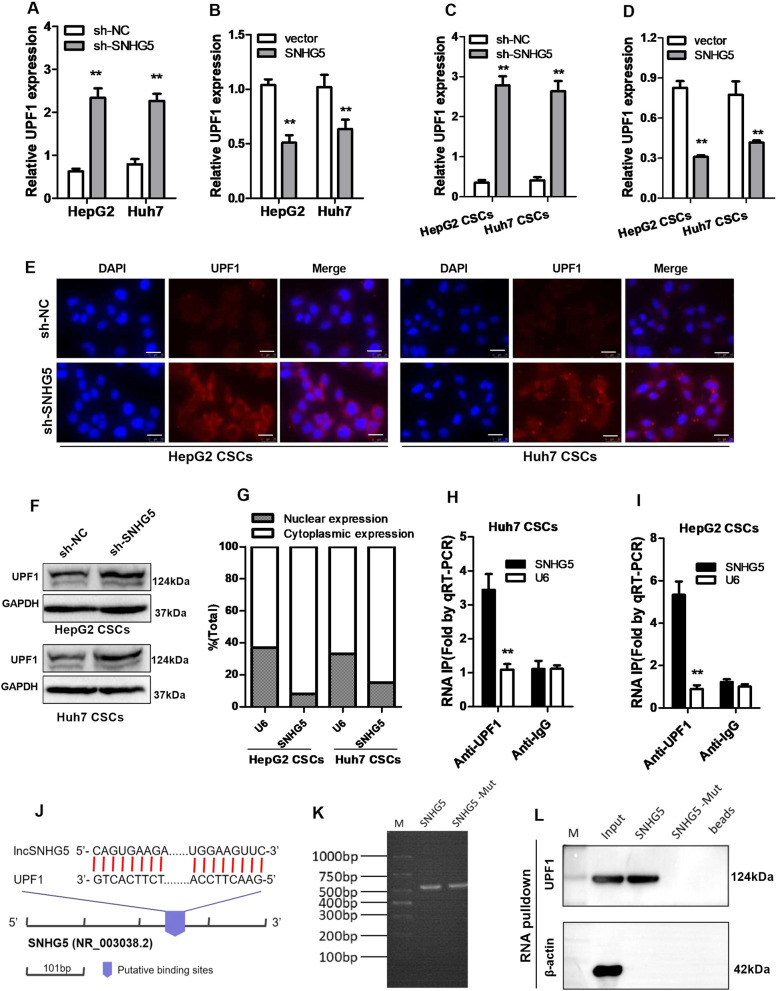
SNHG5 regulates the expression of UPF1. **A**, **B** qRT-PCR analysis of UPF1 expression following transfected HepG2 and Huh7 cells with SNHG5-shRNA or pCMV–SNHG5 vector. **C**, **D** qRT-PCR analysis of UPF1 expression in HepG2 and Huh7 CSCs after downregulation or upregulation of SNHG5. **E** IF analysis of the UPF1 expression in sh-SNHG5 HepG2 and Huh7 CSCs compared with sh-NC cells. **F** Western blot analysis detected the UPF1 protein levels in HepG2 and Huh7 CSCs when SNHG5 was knocked down. GAPDH as a loading control. **G** Cellular localization of SNHG5 in HCC CSCs, U6 as control. **H**, **I** HCC CSC cellular lysates were used for RNA immunoprecipitation (RIP) with UPF1 antibody. Detection of SNHG5 using qRT-PCR. **J** The schematic diagram of binding sites between SNHG5 and UPF1, data from RBPsuite. **K** Gel electrophoresis of SNHG5 wild-type and mutant probes. **L** RNA-pulldown assay was performed to detect the interaction between SNHG5 and UPF1. **compared with sh-NC or vector group, *P* < 0.01. Scale bar: 50 μm.

Recently, several studies have found the role of RNA‐binding proteins (RBPs) in lncRNA-related pathway. In order to identify the proteins that are associated with SNHG5, the starBase and RBPsuite database were used for biological information prediction. The result showed that SNHG5 contains potential binding sites for UPF1. The expression of UPF1 in HCC tissues and healthy tissues from TCGA using GEPIA2 shows that UPF1 expression is reduced in HCC tissues (Figure S2). In order to confirm the regulatory relationship between SNHG5 and UPF1, we first tested the expression of UPF1 in HCC cells after SNHG5 knockdown or overexpression. The result showed that the expression of UPF1 increased in HCC cells and HCC CSCs with depletion of SNHG5 (Fig. 3A, C), while overexpression of SNHG5 significantly inhibited the expression of UPF1 (Fig. 3B, D). Consistently, Western blot and IF assay were performed to detect the expression of UPF1 in liver CSCs, revealing that knockdown of SNHG5 promotes the expression of UPF1 (Fig. 3E, F). To further investigate the interaction between SNHG5 and UPF1, first, we evaluated the cellular orientation of SNHG5 in HCC CSCs. Nuclear and cytoplasmic segments were gained from HCC CSCs. Then, RNA was extracted independently. SNHG5 was discovered mainly in the cytoplasmic fraction (Fig. 3G). Since UPF1 directly binds to SNHG5, we performed RNA immunoprecipitation (RIP) with UPF1 antibody, and observed an enrichment of SNHG5 with UPF1 antibody as compared with the nonspecific antibody (IgG control) (Fig. 3H, I). To further validate the interaction between SNHG5 and UPF1, we obtained the binding sites of SNHG5 and UPF1 through the RBPsuite database. RBPsuite is a database for predicting the binding sites of RNA and RBP. RBPsuite first divides the RNA sequence into 101-nucleotide fragments and scores the interaction between the fragments and RBP. According to the prediction results of the database, the binding site of SNHG5 fragment 3 and UPF1 scored the highest (Fig. S3), and we showed the schematic diagram in Fig. 3J. Therefore, we constructed SNHG5-wild-type(WT) and SNHG5-mutant (Mut) RNA probes (Fig. 3K) and performed RNA-pulldown experiments, the pullsown result showed that UPF1 binds to SNHG5 in HCC CSCs (Fig. 3L).These observations suggested that SNHG5 binds to UPF1 and inhibits UPF1 expression in HCC CSCs.

### Downregulation of UPF1 increases liver CSC-like properties

**Fig. 4:**
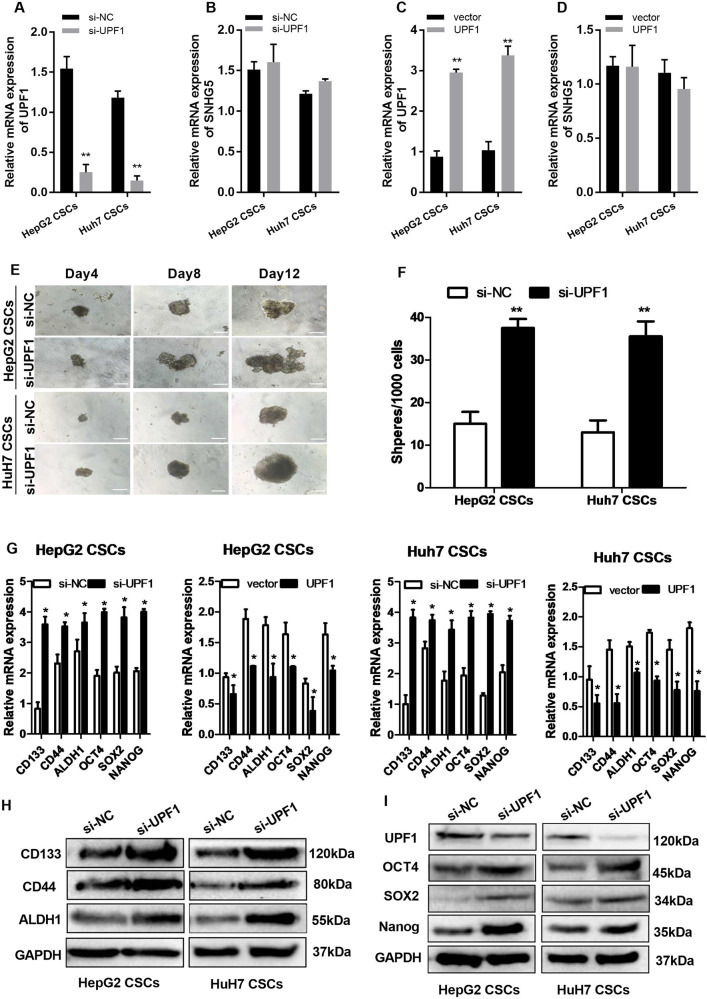
UPF1 is necessary for maintaining HCC CSC proliferation and self-renewal. **A**, **B** qRT-PCR analysis of UPF1 and SNHG5 expression after UPF1 knockdown. **C**, **D** qRT-PCR analysis of UPF1 and SNHG5 expression after UPF1 overexpression. **E** Bright-field microscopy images of the typical morphological features of small aggregates and spheres after silencing of UPF1 in HepG2 and Huh7 CSCs separately on the 4th, 8th, or 12th day, white bar: 50 mm. White bar: 20 mm. **F** Quantification of the total number of primary spheres per 1000 single CSCs after UPF1 knockdown. **G** qRT-PCR analysis of the expression of stem factors (SOX2, OCT4, and NANOG) and markers (CD133, CD44, and ALDH1) in UPF1 knockdown and overexpression of HepG2 and Huh7 CSCs. **G** Western blotting analysis of the expression of stem factors and markers in si-UPF1 HepG2 and Huh7 CSCs compared with si-NC cells. GAPDH as a loading control. *compared with sh-NC or vector group <0.05, **P < 0.01.

We report that SNHG5 suppressed the expression of UPF1, however, it was unclear whether UPF1 regulates SNHG5 expression. First, we depleted the expression of UPF1 by a specific siRNA against UPF1 gene transcript, and upregulated UPF1 by pCMV–UPF1 vector in HepG2 and Huh7 CSCs. The results of qRT-PCR showed that the expression of UPF1 dramatically decreased after transfection with siRNA–UPF1 (Fig. 4A), while pCMV–UPF1 vector increased the expression of UPF1 in HepG2 and Huh7 CSCs compared with the empty vector (Fig. 4C). However, it was interesting that neither downregulation or upregulation of UPF1 did not affect the expression of SNHG5(Fig. 4B, D). This indicated that UPF1 is a downstream gene of SNHG5, and SNHG5 regulates UPF1 in one direction. To explore the role of UPF1 in liver CSC properties, we performed sphere-formation assays, and the result showed that knockdown of UPF1 promoted the liver CSC-like properties of HCC cells (Fig. 4E), the number of spheres per 1000 single-liver CSCs increased after UPF1 downregulation (Fig. 4F). Additionally, qRT-PCR showed that the expression of CSC markers (ALDH1, CD44, and CD133) and stem factors (NANOG, OCT4, and SOX2) was remarkably upregulated with depletion of UPF1, while overexpression of UPF1 showed the opposite effect (Fig. 4G). Meanwhile, this result was verified in Western blot analysis (Fig. 4H, I). These data ultimately suggested that knockdown of UPF1 enhances liver CSC properties.

### Wnt/β-catenin pathway is responsible for liver CSC-like properties

**Fig. 5:**
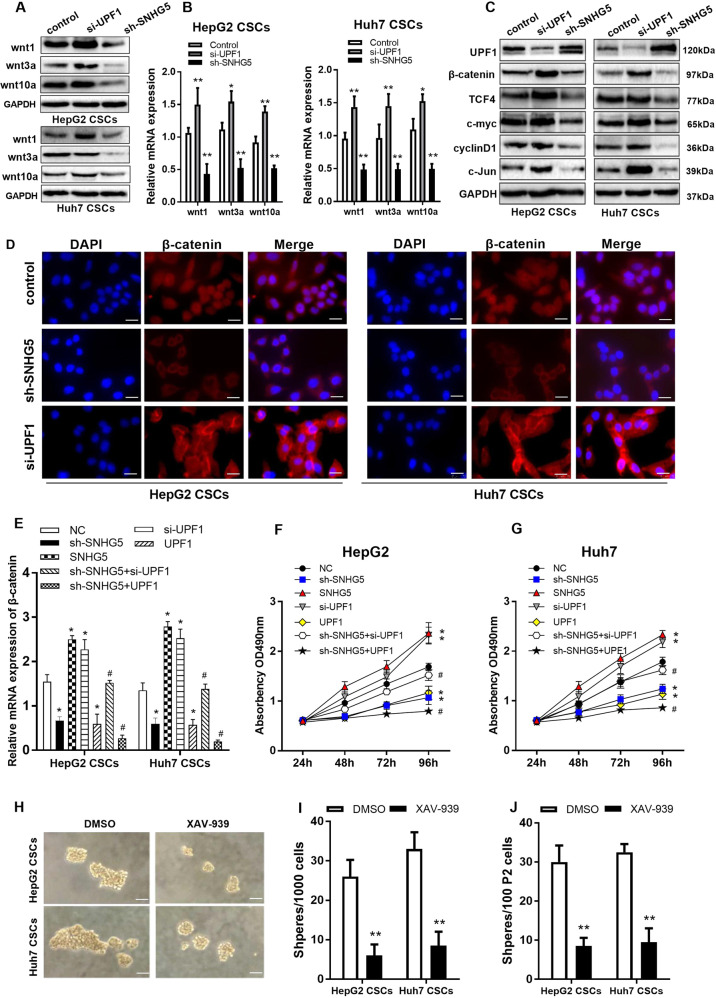
SNHG5/UPF1 axis enhanced CSC properties through the Wnt/β-catenin pathway. **A** Western bloting analysis of the protein levels of Wnt family members (wnt, wnt3a, and wnt10a) in sh-SNHG5, si-UPF1 and control cells. **B** qRT-PCR analysis of the mRNA levels of Wnt family members (wnt, wnt3a, wnt10a) in sh-SNHG5, si-UPF1 and control cells. **C** Western bloting analysis of the protein levels of UPF1 and the key factors of Wnt/β-catenin pathway (β-catenin, c-Jun, TCF-4, cyclinD1, and c-myc) in HepG2 and Huh7 CSCs. GAPDH as a loading control. **D** β-catenin immunofluorescence of seeded HCC CSCs.White bar: 50 mm. **E** qRT-PCR analysis of the mRNA levels of β-catenin in SNHG5- and UPF1-treated cells. **F**, **G** MTT assay detected the cell proliferation of SNHG5- and UPF1-treated cells. **H** Bright-field microscopy images showed the number of spheres derived from Wnt inhibitor XAV-939 at the 12th day of sphere-formation culture. XAV-939 reduced sphere formation. Scale bar: 20 μm. **I**, **J** The effect of Wnt inhibitor on sphere-formation ability was evaluated by counting and comparing the total number of spheres. DMSO served as negative control. *compared with control group <0.05, **P < 0.01. ^#^compared with sh-SNHG5 group <0.05.

**Fig. 6:**
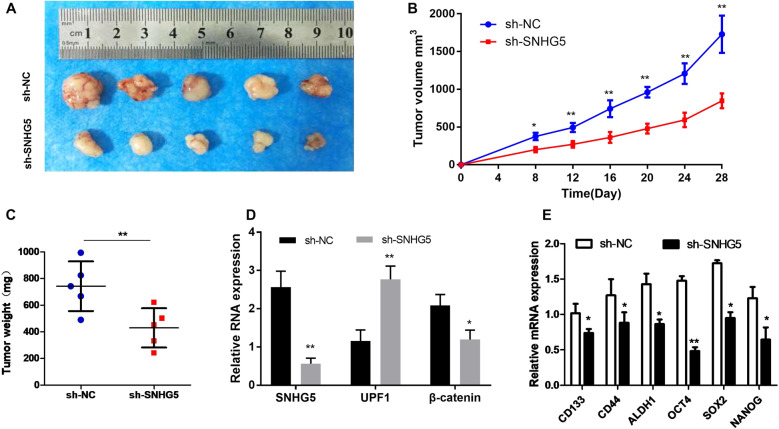
Knockdown of SNHG5 inhibits tumor growth in vivo. **A** The images of formed tumors that were subcutaneously injected with SNHG5–shRNA and NC-shRNA cells. Effect of SNHG5 knockdown on HCC growth in vivo according to the tumor growth curve (**B**) and tumor weight (**C**). In total, 5 mice in each group, and the subcutaneous formatted tumor nodes were harvested after 4 weeks. *P < 0.05; **P < 0.01. **D** The expression of SNHG5, UPF1, and β-catenin in xenograft tumors was detected by qRT-PCR. **E** The expression of CSC markers and stem factors in xenograft tumors were detected by qRT-PCR. *compared with sh-NC group <0.05, **P < 0.01.

Our previous studies have demonstrated a relationship between SNHG5 and the Wnt/β-catenin signaling pathway [14], which is an important pathway for some stem cells. Hence, we speculated that Wnt/β-catenin pathway may be a key factor to liver CSC-like properties. To examine the speculation, we detected the Wnt-family members in HepG2 CSCs and Huh7 CSCs. The result showed that the expressions of Wnt1, Wnt3a, and Wnt10a were downregulated with depletion of SNHG5, and were upregulated with knockdown of UPF1 (Fig. 5A). The result of qRT-PCR also showed the same trend (Fig. 5B). Additionally, the critical components of Wnt/β-catenin pathway (TCF4, c-myc, cyclinD1, and c-Jun) were detected in HepG2 CSCs and Huh7 CSCs by Western blot. We observed that these key components were downregulated following knockdown of SNHG5, while the expression level was increased with depletion of UPF1 (Fig. 5C). It is known that the β-catenin is a key factor of Wnt/β-catenin pathway. Therefore, we detected the expression of β-catenin in HepG2 CSCs and Huh7 CSCs with IF analysis. The result illustrated that the nucleic β-catenin protein expression amplified in liver CSCs with depletion of UPF1, while SNHG5 knockdown attenuated the level of nucleic β-catenin (Fig. 5D).To better prove the rationality of the SNHG5/UPF1 signal axis, we performed rescue (adding-back) experiment, that is, to detect whether si-UPF1 can restore the sh-SNHG5 cell phenotype. The result of qRT-PCR showed that knockdown of SNHG5 significantly inhibited the expression of β-catenin, but downregulating UPF1 can partially restore the inhibitory effect of sh-SNHG5 on β-catenin expression (Fig. 5E). At the same time, MTT experiment revealed that downregulating UPF1 can partially restore the inhibitory effect of sh-SNHG5 on HCC cell proliferation, while UPF1 vector intensified the inhibitory effect of sh-SNHG5 (Fig. 5F, G). To further validate the function, XAV-939, a Wnt/β-catenin pathway inhibitor was adopted. With XAV-939, the sphere formation of liver CSCs was impaired and the number of spheres per 1000 single liver CSCs decreased (Fig. 6H–J). These results suggested that SNHG5 regulates the activation of the Wnt pathway through UPF1.

### Knockdown of SNHG5 represses tumor growth in vivo

**Fig. 7:**
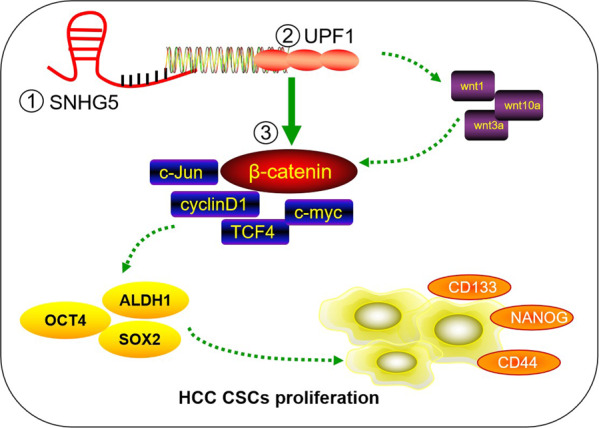
A schematic model depicting the functions of SNHG5 during the proliferation and cancer stem cell-like properties of HCC. SNHG5 promotes the proliferation and cancer stem cell-like properties of HCC by regulating UPF1 and activating Wnt-signaling pathway.

HepG2 and Huh 7 cells transfected with SNHG5–shRNA and negative control(NC) were subcutaneously injected into male nude mice for 5 weeks. Tumor growth curve revealed that HCC cells transfected with SNHG5–shRNA greatly inhibited tumor growth compared with NC group (Fig. 6A). We also observed the tumor volume and tumor weight among the two groups. The results showed that downregulation of SNHG5 suppressed tumor volume and weight, therefore inhibiting the tumor growth effectively (Fig. 6B, C). Additionally, the expression of SNHG5 and UPF1 was detected in xenograft tumors. The result of qRT-PCR indicated that the expression of SNHG5 greatly decreased, while the expression of UPF1 greatly increased in SNHG5–shRNA xenograft tumors (Fig. 6D). To test all their results at the same baseline level, we verified the expression levels of β-catenin, CSC markers, and stem factors in animal tumor tissues after knockdown of SNHG5 by qRT-PCR. The results showed that the expression levels of β-catenin, CSC markers and stem factors were decreased compared with the control group (Fig. 6D, E), which is also consistent with the results obtained in in vitro experiments. In summary, this study basically confirmed that SNHG5 promotes HCC proliferation and cancer stem cell-like properties by regulating UPF1 to activate the Wnt-signaling pathway (Fig. 7).

## Discussion

CSCs are a subgroup of cells with self-renew and differentiation properties, which have been isolated in many solid cancers [18–20]. Liver CSCs were proved to contain various subtypes,these were characterized by different surface makers, including CD133^+^CD13^+^, EpCAM^+^CD24^+^OV6^+^, CD133^+^CD44^+^CD24^+^ EpCAM^+^, and so on [21]. CSCs contribute to the epithelial–mesenchymal transition (EMT), metastasis, drug resistance, and radio resistance through varieties of mechanisms [22–24]. Numerous efforts have been made to dissect the molecular mechanisms involved in the regulation of liver CSCs with the intent to identify novel therapeutic strategies to improve the poor prognosis of HCC.

Recently, it has been revealed that lncRNAs played critical roles in CSCs. For example, in glioma, the downregulation of lncRNA–ROR promoted the proliferation of cancer cells and the formation of a sphere of stem cells with the down expression of stem cell factor KLF4 [21]. In the case of HCC, it also has been verified that many lncRNAs are responsible to drive CSC self-renewal and tumor progression through various mechanisms [25, 26]. Based on this, lncRNA is expected to become an important therapeutic agent for HCC. The diverse functional repertoire of lncRNAs reveals various opportunities for their therapeutic targeting, the means of which need to be adjusted to the mode of action of the lncRNA [27]. Battistelli C et al. [28] designed a HOTAIR deletion mutant form, named HOTAIR-sbid, which was proven to reduce cellular motility, invasiveness, anchorage-independent growth, and responsiveness to TGFβ-induced EMT. These data provide evidence on a lncRNA-based strategy to effectively impair tumor metastases. Although studies have confirmed that lncRNAs function as critical regulators of gene expression in embryonic and induced pluripotent stem cells, the previous understanding of their role in CSCs has been limited.

SNHG5 has been widely proven to play an important role in a variety of tumors, such as colorectal cancer, gastric cancer and osteosarcoma, and even myeloid leukemia [29–32]. In the present study, we unraveled a critical role for SNHG5 in liver CSC properties,SNHG5 alterations (knockdown or upregulation) significantly influenced the proliferation and self-renewal capacity of liver CSCs. Similarly, the expression of stem cell markers and stem factors decreased after downregulation of SNHG5. Our findings provided important insights into the relationship between SNHG5 and liver CSCs.

In order to further investigate the mechanism of SNHG5 regulating the properties of liver CSCs, bioinformatic methods were used to find the potential target genes of SNHG5. We found that the key factor of nonsense-mediated mRNA decay (NMD) UPF1 contains a potential binding site with SNHG5.UPF1 plays a critical role in RNA degradation pathways, and also promotes the decay of mRNAs encoding many other proteins that oppose the proliferative, undifferentiated cell state [33]. UPF1 acts, in part, by destabilizing the NMD substrate encoding the TGFβ inhibitor, Smad7, and stimulating TGF signaling [34], and several studies have shown that UPF1 exerts suppressive roles in tumor progression [35]. As expected, UPF1 inhibited the stemness of liver CSCs. Mechanistically, we found that SNHG5 combined with UPF1, and the overexpression of SNHG5 following the downregulation of UPF1 with the downexpression of surface makers and stem factors. These results revealed that SNHG5 promotes the proliferation and cancer stem cell-like properties of HCC by regulating UPF1. However, the detailed binding sites within SNHG5 with UPF1 are still unclear, this needs to be further studied.

Recently, several researches demonstrated that Wnt/β-catenin signaling played a critical role in cancer stem cells. For example, in glioma, the self-renewal and tumorigenicity of CSCs were regulated by dysregulated Wnt–FoxM1/β-catenin signaling pathway [36]. However, its role in liver CSCs was not completely known. Our data indicate that inhibition of the Wnt pathway results in an obviously impaired sphere formation capacity. We have also demonstrated that UPF1 is responsible for liver CSC characteristics, UPF1 mediates the activation of the Wnt pathway in liver CSCs by regulating the expression of Wnt, and SNHG5 mediated the activation of the Wnt pathway in liver CSCs by regulating UPF1 expression.

In summary, we conclude that SNHG5 promoted HCC cell proliferation in vitro and in vivo, and was responsible for the sphere formation of liver CSCs and the CSC properties. The underlying mechanism of SNHG5 promoting the proliferation and CSC-like properties of HCC was by regulating UPF1 and activation of the Wnt-signaling pathway. Our current data imply that SNHG5, along with its downstream mechanism and pathways, could shed light on new potential therapeutic targets against liver CSCs.

## Supplementary information

Supplementary figures

Supplementary tables
