# Supplementary material for: LncRNA SNHG5 promotes the proliferation and cancer stem cell-like properties of HCC by regulating UPF1 and Wnt-signaling pathway
Source: Cancer Gene Ther. 2022 Mar 25;29(10):1373–83. doi: 10.1038/s41417-022-00456-3 (PMC9576592; doi:10.1038/s41417-022-00456-3)
Supplement: Supplementary file 1 — Supplementary figures [file 41417_2022_456_MOESM1_ESM.docx]

Supplementary figures


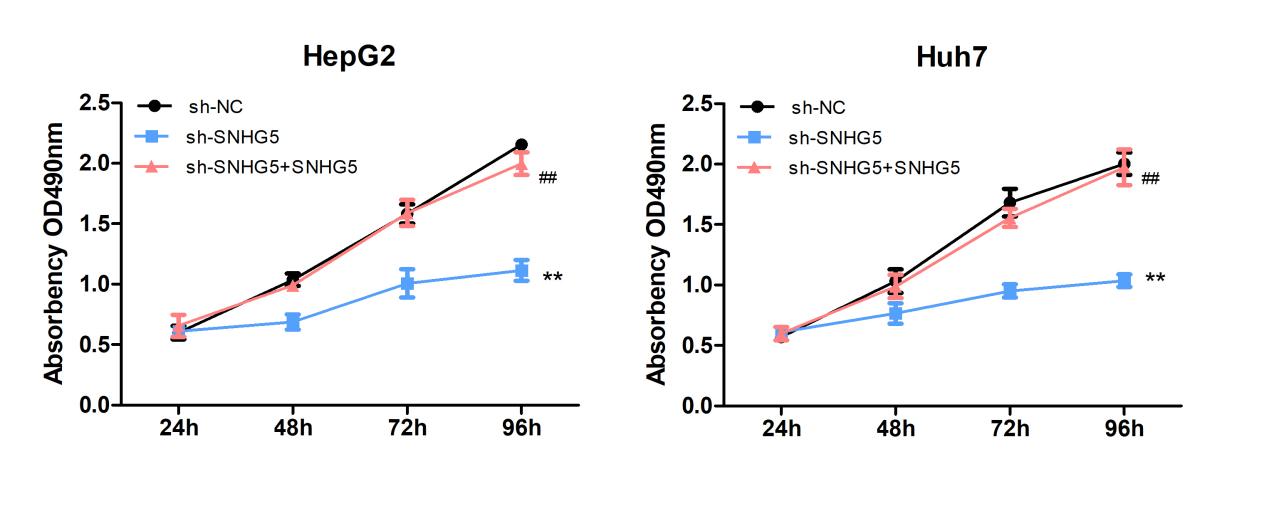


Figure S1. MTT assay showed that SNHG5 vector can restore the inhibition of sh-SNHG5 on cell proliferation.


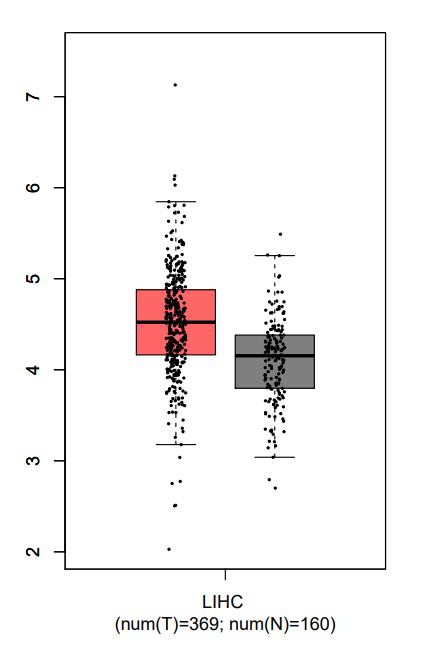


Figure S2. The expression of UPF1 in HCC tissues and healthy tissues from TCGA using GEPIA2 shows that UPF1 expression is reduced in HCC tissues


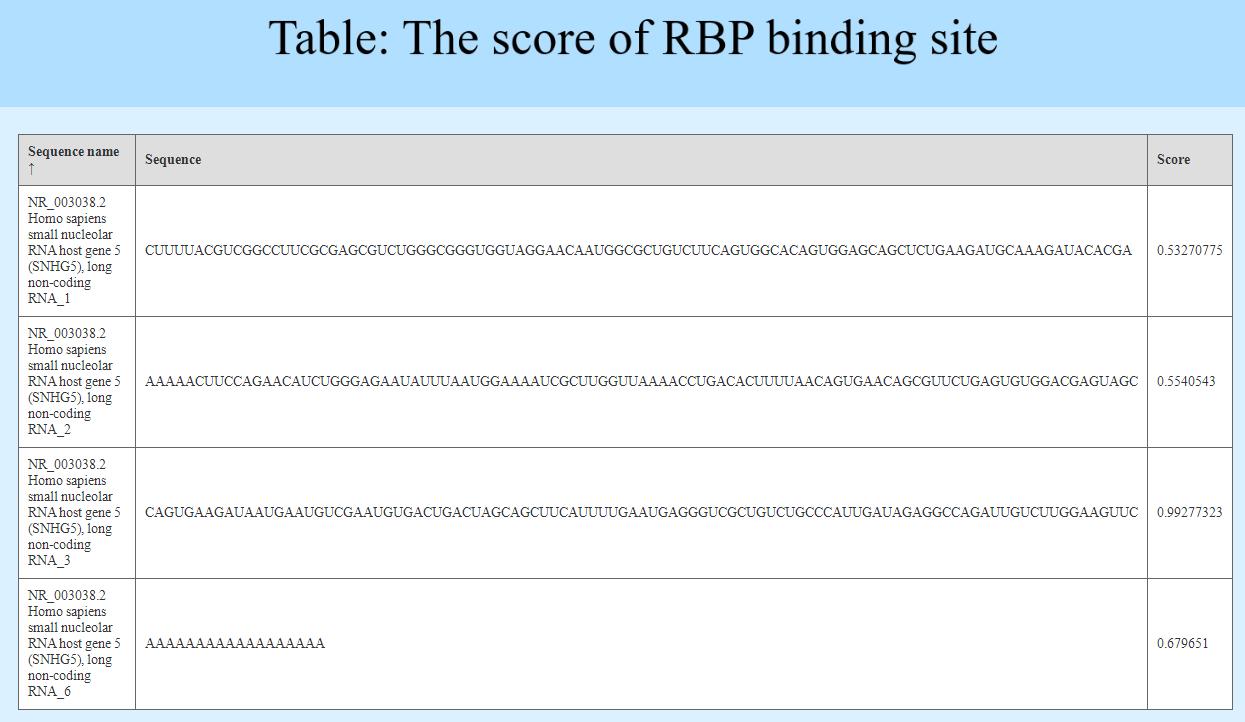


Figure S3. The predicted binding site of SNHG5 on UPF1 by RBPsuite.
