# Supplementary material for: LncRNA SNHG5 promotes the proliferation and cancer stem cell-like properties of HCC by regulating UPF1 and Wnt-signaling pathway
Source: Cancer Gene Ther. 2022 Mar 25;29(10):1373–83. doi: 10.1038/s41417-022-00456-3 (PMC9576592; doi:10.1038/s41417-022-00456-3)
Supplement: Supplementary file 2 — Supplementary tables [file 41417_2022_456_MOESM2_ESM.pdf]

Supplementary Tables

Supplementary Table 1. Primer sequences and target sequences used in this study

Gene	Sequence or Target Sequence
SNHG5-F	5'-CGAGTAGCCAGTGAAGATAATG-3'
SNHG5-R	5'-CACACAACAGTCAAGTAAACC-3'
β-actin-F	5'-ATCGTGCGTGACATTAAGGAGAAG-3'
β-actin-R	5'AGGAAGGAAGGCTGGAAGAGTG-3'
UPF1-F	5'-ACCACGAAGTTGCTGAAGG-3'
UPF1-R	5'-ACACAGGACAGGATGATGAAG-3'
Wnt1-F	5'-ATTCTGATTCTGCTGCTCTTG-3'
Wnt1-R	5'-AGTCCTGGTCCTCTTCTCC-3'
Wnt3a-F	5'-CATCATCCTGCTTATCCTTGTG-3'
Wnt3a-R	5'-CATAGTCCTGGTCTTCTTCTCC-3'
Wnt10a-F	5'-GCTGGAAGGCGAGGCGAGGAGAG-3'
Wnt10a-R	5'-CAACCGTCTTAATCAGAAGTGTC-3'
OCT4-F	5'-TATGCAAAGCAGAAACCCTCGTG-3'
OCT4-R	5'-CTGGCGCCGGTTACAGAACCA-3'
SOX2-F	5'-ATGACCAGCTCGCAGACCTAC-3'
SOX2-R	5'-TTGACCACCGAACCCATGGAG-3'
NANOG-F	5'-TCCAGCAGATGCAAGAACTCTCCA-3'
NANOG-R	5'-CACACCATTGCTATTCTTCGGCCA-3'
CD133-F	5'-GGTCTGGCGAGCTAAGGGAA-3'
CD133-R	5'-GGGGAAGGCAAGCGTGTT-3'

CD44-F	5'-TTTGCATTGCAGTCAACAGTC-3'
CD44-R	5'-GTTACACCCCAATCTTCATGTCCAC-3'
ALDH1-F	5'-GTTCCTGGTTATGGGCCTAC-3'
ALDH1-R	5'-CCTGGATGCGGCTATACAAC-3'
SNHG5-shRNA	5'-CAGUGAAGAUAAUGAAUGUTT-3'
SNHG5-shRNA	3'-ACAUUCAUUAUCUUCACUGTT-5'
UPF1-siRNA-F	5'-CAGUGAAGAUAAUGAAUGUTT-3'
UPF1-siRNA-R	3'-ACAUUCAUUAUCUUCACUGTT-5'
siRNA-NC-F	5'-UUCUCCGAACGUGUCACGUTT-3'
siRNA-NC-R	3'-ACGUGACACGUUCGGAGAATT-5'

Supplementary Table 2. Antibodies used in this study

Antibody (Item No.)	Specificity		Company	
	WB	IF		
GAPDH (D16H11)	1:1000		Rabbit monoclonal	Cell Signaling Technology
UPF1(EPR4681)	1:1000	1:500	Rabbit monoclonal	Abcam
β-catenin(D10A8)	1:1000	1:500	Rabbit monoclonal	Cell Signaling Technology
CyclinD1(EPR2241)	1:1000		Rabbit monoclonal	Abcam
c-Myc(D3N8F)	1:1000		Rabbit monoclonal	Cell Signaling Technology
C-Jun(EP693Y)	1:1000		Rabbit monoclonal	Abcam
TCF4(C48H11)	1:1000		Rabbit monoclonal	Cell Signaling Technology
Oct-4A (C52G3)	1:1000		Rabbit monoclonal	Cell Signaling Technology
Sox2 (D6D9)	1:500		Rabbit monoclonal	Cell Signaling Technology
Nanog (D2A3)	1:1000		Rabbit monoclonal	Cell Signaling Technology
CD133 (D2V8Q)	1:500	1:300	Rabbit monoclonal	Cell Signaling Technology
CD44 (156-3C11)	1:1000	1:500	Mouse monoclonal	Cell Signaling Technology
ALDH1 (D9J7R)	1:1000	1:500	Rabbit monoclonal	Cell Signaling Technology
